# Characterization and Design of Three-Phase Particulate Composites: Microstructure-Free Finite Element Modeling vs. Analytical Micromechanics Models

**DOI:** 10.3390/ma16186147

**Published:** 2023-09-10

**Authors:** Sebak Oli, Yunhua Luo

**Affiliations:** Department of Mechanical Engineering, University of Manitoba, Winnipeg, MB R3T 2N2, Canada

**Keywords:** three-phase particulate composites, microstructure-free, finite element modeling, elastic property, analytical models, accuracy comparison

## Abstract

Three-phase particulate composites offer greater design flexibility in the selection of phase materials and have more design variables than their two-phase counterparts, thus providing larger space for tailoring effective properties to meet intricate engineering requirements. Predicting effective elastic properties is essential for composite design. However, experimental methods are both expensive and time intensive, whereas the scope of analytical micromechanics models is limited by their inherent assumptions. The newly developed microstructure-free finite element modeling (MF-FEM) approach has been demonstrated to be accurate and reliable for two-phase particulate composites. In this study, we investigate whether the MF-FEM approach can be applied to three-phase particulate composites and, if applicable, under which conditions. The study commences with a convergence analysis to establish the threshold ratio between the element size and the RVE (representative volume element) dimension. We then validate the MF-FEM approach using experimental data on three-phase composites from the existing literature. Subsequently, the MF-FEM method serves as a benchmark to assess the accuracy of both traditional and novel analytical micromechanics models, in predicting the effective elasticity of two distinct types of three-phase particulate composites, characterized by their small and large phase contrasts, respectively. We found that the threshold element-to-RVE ratio (1/150) for three-phase composites is considerably smaller than the ratio (1/50) for two-phase composites. The validation underscores that MF-FEM predictions align closely with experimental data. The analytical micromechanics models demonstrate varying degrees of accuracy depending on the phase volume fractions and the contrast in phase properties. The study indicates that the analytical micromechanics models may not be dependable for predicting effective properties of three-phase particulate composites, particularly those with a large contrast in phase properties. Even though more time-intensive, the MF-FEM proves to be a more reliable approach than the analytical models.

## 1. Introduction

Three-phase composites have several advantages over two-phase composites. They offer greater design flexibility and exhibit superior mechanical properties compared to two-phase composites. Different hybrid metal–matrix composites are receiving attention in the aerospace and automotive industries due to their high strength, low density, and enhanced ductility and toughness. For example, alloy Al 6063 with 90 wt% Al, 5wt% SiC, and 5 wt% Gr exhibits superior mechanical properties compared to the Al 6063 variant with 90 wt% Al and 10 wt% SiC. The former demonstrates a higher tensile strength of 190.48 MPa and a lower density of 2.64 g/cc, while the latter shows a lower tensile strength of 160.84 MPa and a higher density of 2.71 g/cc [1]. Similarly, the TiC/TiB_2_/Al composite eliminates defects such as interfacial discontinuity and macro pores observed in the TiC/Al composite. This elimination leads to significant improvements in its yield strength, ultimate compressive strength, and plastic strain [2].

A critical task in the design of composite materials is to predict their macroscopic properties based on the microstructure information. There are basically three types of approaches available for the prediction, i.e., analytical, experimental, and numerical. The experimental approach is usually used for validation but it is costly if applied at every intermediate design. The use of numerical methods has significantly increased in recent years due to their computational advances, but analytical models remain the most effective methods in terms of both time and cost. However, the accuracy of analytical methods is largely affected by the adopted assumptions, usually regarding inclusion property, geometry, quantity, and distribution. These assumptions are made to reduce the complexity so that the derivation of analytical solutions is possible. Consequently, their applicability depends on the satisfaction of the adopted assumptions. Moreover, some models are intended to determine the lower and upper bounds rather than exact estimations [3,4,5].

Over the past few decades, several micromechanics models have been developed to estimate the elastic properties of composites which were later either improved or extended for multiphase composites by several researchers. In 1965, two researchers, Budiansky [6] and Hill [7], independently introduced self-consistent (SC) models in which a single inclusion is considered to be embedded directly into the effective composite media of unknown properties. Because of this assumption, they are well-known for overestimating the effective moduli of composites at higher inclusion volume fractions, and later an improved version, the generalized self-consistent (GSC) model [8,9,10], was proposed for two-phase composites. The model considers a single inclusion surrounded immediately by a matrix phase and then the whole structure is embedded into an equivalent composite media. This new approach, also known as three-phase model or inclusion-matrix-composite model, was able to overcome the drawbacks of the self-consistent method by considering the matrix-inclusion interactions.

Many researchers have contributed to extending the applicability of the classical GSC model of two-phase into multiphase composites. In 1986, Benveniste [11] presented the embedding procedure of the GSC model into multiphase media having spherical inclusions and later Siboni and Benveniste in 1991 [12] extended the idea for a more complex context and generalized it to multiphase particulate and fibrous composite. Huang and his colleagues [13] used the equivalent energy approach of Budiansky and extended the GSC model developed by Christensen and Lo (1979) [10] to multiphase composites and established that the dependence on individual phases can be decoupled into multiple two-phase composites. Furthermore, they also provided several variations in the GSC method for hybrid composites (three-phase) [14].

Another approach that is different from the GSC method is the Mori–Tanaka (MT) method [15] which neglects the interactions among inclusions and involves the evaluation of average strain within an isolated inclusion embedded in an infinite matrix subjected to the average matrix stress. This method produces a closed-form solution and is uniquely linked to the well-known Hashin–Shtrikman (HS) bounds [5]. Based on the MT concept of average stress in the matrix, Weng in 1984 [16] presented a method to obtain elastic properties of multiphase composites. Furthermore, Mares [17] evaluated three different models for a three-phase composite namely the Paul model, the Paul estimation for upper and lower bounds, and the Halpin-Tsai model having closed-from expression. Paul’s estimation for bounds is essentially the Voigt–Reuss (VR) expressions for three-phase.

A number of papers with varying applications are available for obtaining elastic properties of the three-phase particulate composite, but the study of their effectiveness based on the inclusion’s volume content is not widely studied due to the lack of complete experimental data. Validation with experimental data is considered an ideal way to determine the accuracy of different models, but the availability of suitable experiments is always a scarce resource, and it is even rarer for a three-phase composite. It is also worth mentioning that they are not always free from various forms of experimental defects and human errors. To overcome such difficulties, Luo developed a microstructure-free finite element modeling (MF-FEM) approach [18,19], and applied it to perform an accuracy comparison among selected micromechanics models for two-phase composites. MF-FEM shows nice agreement with experimental data and has several advantages over microstructure-based finite element modeling (MB-FEM). In MB-FEM, acquiring all microstructural details—such as inclusion shape, size, and distribution—of the composite is essential for constructing the finite element model. Not only is this process time-consuming, but it can also result in an ill-conditioned finite element mesh. While in MF-FEM, as the name suggests, actual microstructural details are not required in the model during the simulation which eliminates the tedious task of creating microstructure geometry. Inclusions are represented by brick elements which always results in high-quality mesh. While the accuracy and reliability of MF-FEM have been established for two-phase particulate composites [18,19], its applicability to three-phase composites remains uncertain. This paper aims to investigate whether MF-FEM can be extended to three-phase composites and, if so, under what conditions.

## 2. Microstructure-Free Finite Element Modeling of Three-Phase Particulate-Composites

The Microstructure-Free Finite Element Modeling (MF-FEM) approach assumes that the effective elastic properties of the representative volume element (RVE) remain independent of the shape and size of the inclusions, provided the inclusions are sufficiently smaller than the RVE. This versatility allows inclusions to be represented by elements of any shape and size [19], making it particularly advantageous for designing particulate composites. For two-phase composites, the inclusion size is typically limited to fifty times smaller than the RVE size [19]. In this section, we determine the threshold inclusion size for three-phase particulate composites, by varying the inclusion-to-RVE size ratios to observe convergence of RVE effective properties. We consider two types of three-phase composites with small and large contrasts in their elastic properties, as presented in Table 1.

**Figure 1:**
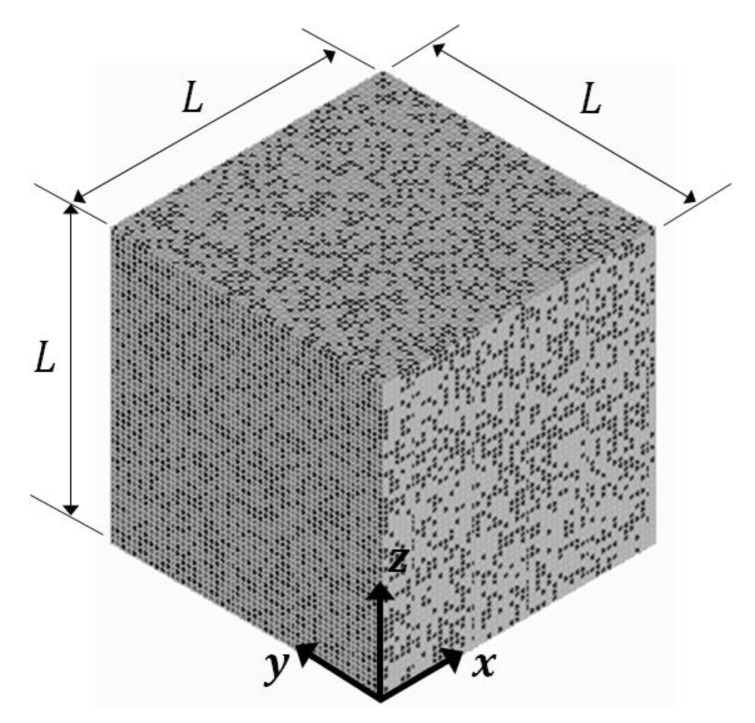
Composite RVE and coordinate system.

More details of the study are provided below:

- The two composites having the volume fractions as described in Table 1 will be referred to as SPC303535 and LPC303535, respectively.
- All other parameters are kept constant in the study, except the size of elements.
- The starting element-to-RVE size ratio is 1/50, which was determined as the threshold ratio for two-phase particulate composites [19]. However, in the case of three-phase composites, the inclusion-to-RVE size ratio is anticipated to be smaller.
- Commercial software ANSYS Mechanical APDL (2021 R1) is used for this study. The cubic RVE shown in Figure 1 has side length L=100, it is uniformly meshed using brick element SOLID185 (with reduced integration), all the elements have the same size.
- Effective Young’s modulus and Poisson’s ratio of the RVE are characterized by MF-FEM. Bulk modulus and shear modulus are calculated using the elasticity relations.
- Boundary conditions listed in Table 2 are applied in the characterization. As has been demonstrated in two-phase particulate composites [18,19], the differences in the effective properties characterized in x, y, and z directions will become smaller and smaller with reduced element size. The presented results are the averages of values in the three directions.

**Table 2:** RVE boundary conditions for the characterization of composite effective properties [18].

RVE Surface	Young’s Modulus (*E_i_, i = x, y, z*) and Poisson’s Ratio (*ν_ij_, i, j = x, y, z*)		
	*E_x_, ν_xy_, ν_xz_*	*E_y_, ν_yx_, ν_yz_*	*E_z_, ν_zx_, ν_zy_*
*x* = 0	*u_x_* = 0	*u_x_* = 0	*u_x_* = 0
*y* = 0	*u_y_* = 0	*u_y_* = 0	*u_y_* = 0
*z* = 0	*u_z_* = 0	*u_z_* = 0	*u_z_* = 0
*x* = 100	*u_x_* = 1	Homogeneous *u_x_*	Homogeneous *u_x_*
*y* = 100	Homogeneous *u_y_*	*u_y_* = 1	Homogeneous *u_y_*
*z* = 100	Homogeneous *u_z_*	Homogeneous *u_z_*	*u_z_* = 1

### 2.1. Threshold of Element-to-RVE Size for Three-Phase Particulate Composites

**Figure 2:**
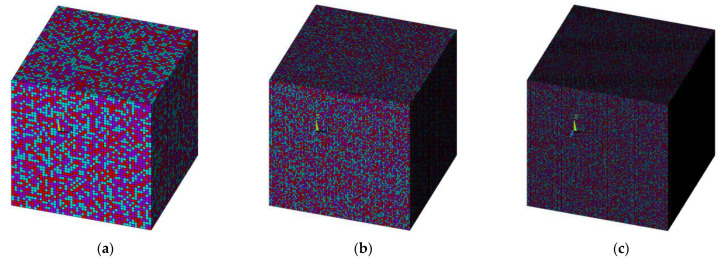
RVE with 30%, 35%, and 35% of softer, stiffer, and intermediate phases (red, blue, purple) for different element-to-RVE size ratios (**a**) 1/50; (**b**) 1/100; (**c**) 1/150.

**Figure 3:**
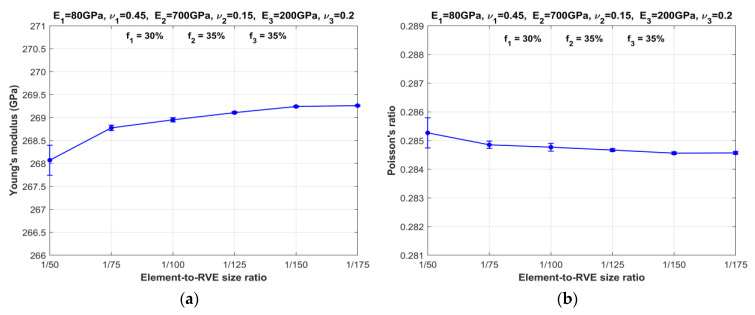
Variation in RVE properties of composite SPC303535 with element-to-RVE size ratio: (**a**) Young’s modulus; (**b**) Poisson’s ratio.

**Figure 4:**
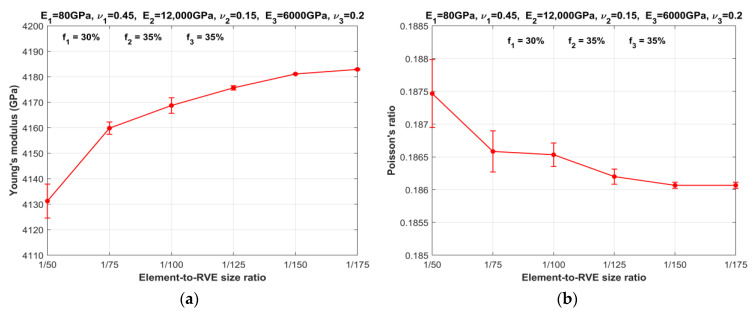
Variation in RVE properties of composite LPC303535 with element-to-RVE size ratio: (**a**) Young’s modulus; (**b**) Poisson’s ratio.

To determine the threshold of element-to-RVE size ratio for three-phase particulate composites, a series of numerical simulations are performed. The number of elements is systematically increased while maintaining a constant RVE size, as depicted in Figure 2. The objective is to observe the influence of particle size, i.e., element size, on the effective properties of three-phase particulate composites characterized by MF-FEM. The results, presented in Figure 3 and Figure 4, respectively, for SPC303535 and LPC303535, clearly demonstrate that reducing the element size leads to converged properties and minimizes anisotropy. As the element size decreases, there is a noticeable reduction in the difference between the Young’s modulus and Poisson’s ratio values of consecutive models. In other words, the change in elastic properties between models with different element sizes becomes less pronounced when transitioning from larger to smaller element-to-RVE size ratios. The graphs show a steady convergence, but it is worth noting that composite LPC303535 exhibits a slightly slower rate of convergence. After careful analysis, a threshold of 1/150 size ratio was chosen for further investigation. This decision was not only driven by computational efficiency but also because the change in elastic properties for both models, when the size ratio reduced from 1/150 to 1/175, remained within 0.1%.

### 2.2. Experimental Validation

The availability of comprehensive experimental data on the elastic properties of three-phase particulate composites covering a wide range of volume fractions is limited. One noteworthy set of experimental data was conducted by Cohen and Ishai [20], where they investigated three-phase composites consisting of an epoxy matrix with quartz-sand fillers and voids. Cohen and Ishai’s experiments encompass testing data for both tension and compression on three different sets of composites, each having a constant filler-to-matrix weight ratio (*n*) of 0.5, 1, or 1.5. For our validation purposes, we specifically utilized the compression data of the composites with a filler-to-matrix weight ratio of *n* = 0.5. To establish a relationship between the volume content of sand (f_1_) and the void content (f_2_) for composites with a filler content of *n* = 0.5, we used the densities listed in Table 3. Consequently, we derived a simple expression for the relationship: f_1_ = 0.173(1 − f_2_).

**Figure 5:**
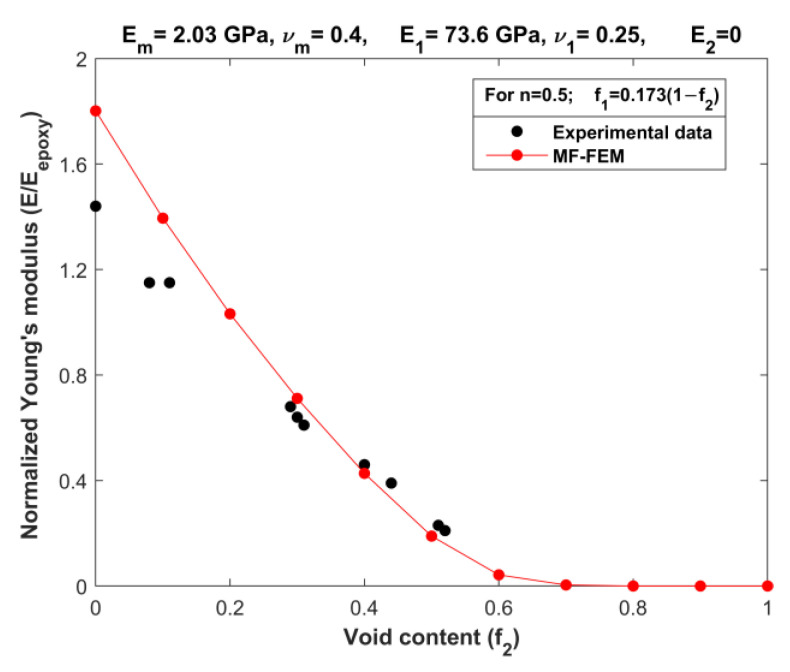
Validation of MF-FEM against experimental data obtained by Cohen and Ishai [20] (weight ratio *n* = 0.5:1).

Figure 5 presents a comparison between the predictions obtained from the MF-FEM of the porous matrix composite and the corresponding experimental data obtained from the work of [20]. Overall, there is a satisfactory agreement between the MF-FEM predictions and the experimental data within the mid-range of the porosity. However, a notable discrepancy becomes apparent in cases of low or negligible porosity, representing the two-phase scenario. The MF-FEM tends to overestimate Young’s modulus values in this region. One likely explanation for this discrepancy is that the actual samples used in the experiment were not entirely free from porosity, unlike the idealized model employed in the MF-FEM simulations. This disparity in porosity levels between the real samples and the model could be a contributing factor to the observed differences in Young’s modulus values in the two-phase zone.

Another set of experimental data utilized for validation comes from Yang [21], which pertains to mortar with a transition zone (TZ). This is viewed as a three-phase composite without voids. Yang’s study focused on the behavior of transition zone and its influence on the elastic modulus of mortar. His research established that the overall modulus of mortar is subjected to the elastic properties and volume content of the TZ, which functions as the third phase between the aggregate and cement paste. Through a comparative analysis of theoretical outcomes and empirical data, Yang inferred that the volume fraction of TZ can be estimated from aggregate volume fraction (*f_a_*) by

(1) $$f_{\mathrm{TZ}}=0.634\;\times \;f_{a}$$

**Figure 6:**
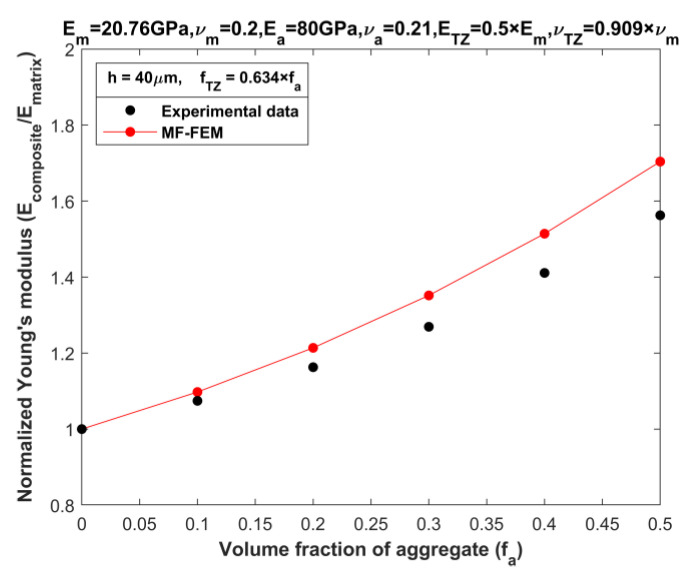
Validation of MF-FEM against experimental data by Yang [22].

The material properties of the three phases in the mortar can be found in Table 4. These properties were also used in MF-FEM to predict effective Young’s modulus of the mortar. Experimental data, as measured by Yang [21], are detailed in Table 5, with the validation results illustrated in Figure 6. The validation results further confirm the reasonable agreement between the MF-FEM predictions and experimental data.

## 3. Comparison between MF-FEM and Analytical Micromechanics Models

**Figure 7:**
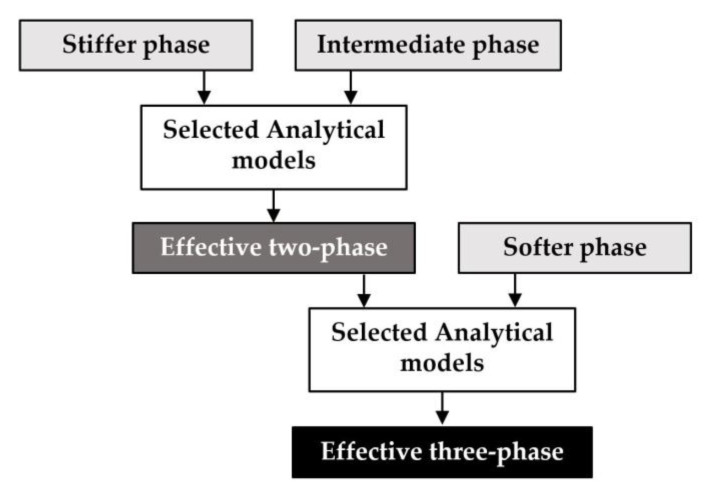
Prediction of effective properties of three-phase composites using analytical micromechanics models.

The analytical models presented below are widely employed for calculating the elastic properties of two-phase particulate composites. Some of the models require a clear identification of the matrix and inclusion phases as input and are extended to predict the properties of three-phase composites by applying expressions between two materials and combining the resulting effective properties with the third material using the same expression. However, the selection of two phases from three materials can be performed in multiple ways, leading to minor discrepancies in the final predictions depending on the initial choice. To minimize the effect of this discrepancy, we maintain consistency in phase selection during the initial calculations. Specifically, we consistently choose stiffer and intermediate phases for all models and combine the resulting two-phase effective properties with the softer phase to obtain the final properties of three-phase composites (see Figure 7). MF-FEM overcomes this limitation by producing a single output regardless of phase type. For the range of phase fractions where experimental data are lacking, we utilize MF-FEM results as a substitute to evaluate and compare the performance of various analytical micromechanics models.

### 3.1. Analytical Micromechanics Models

In this study, we have carefully selected several micromechanics models from the existing literature that are widely employed for predicting elastic properties in two-phase composites. We then applied these models to three-phase composites. Additionally, we have incorporated newly developed models, such as the isotropized Voigt–Reuss model [23] and the Iterative isotropization of VR and HS bounds [24], to further enrich the comparison study. By considering a diverse range of models, we aim to comprehensively evaluate their effectiveness in predicting the elastic properties of three-phase composites.

#### 3.1.1. The Voigt and Reuss (VR) Model [3,4]

The Voigt and Reuss models are fundamental approaches based on the iso-strain and iso-stress concepts, respectively. In the Voigt model, phase materials are assumed to work in parallel, resulting in maximum stiffness, while the Reuss model considers them to work in series, achieving maximum flexibility. These models are commonly utilized in the study of novel composite materials and are capable of providing upper and lower bounds for the elastic properties. By adopting these complementary models, researchers can effectively analyze and understand the potential range of elastic behavior exhibited by the composite material under investigation.

(2) $$P_{V}=f_{0}P_{0}+f_{1}P_{1}+f_{2}P_{2}$$

(3) $$P_{R}=\frac{1}{\frac{f_{0}}{P_{0}}+\frac{f_{1}}{P_{1}}+\frac{f_{2}}{P_{2}}}$$

where P represents any elastic properties of composites and subscript V and R represent Voigt and Reuss formulas, respectively. They are often applied for Young’s modulus calculation; however, both models consider all four elastic properties as independent parameters, they are also widely used for shear modulus, bulk modulus, and Poisson’s ratio estimation.

#### 3.1.2. The Voigt–Reuss-Hill (VRH) Average Model [25]

The Voigt and Reuss models represent two extreme cases, depicting the parallel and series combination of inclusions, respectively. However, due to the inherent anisotropy introduced by these micromechanics models, they cannot precisely estimate the exact properties of the composite material. To address this limitation, the VRH (Voigt–Reuss-Hill) average model, which takes the arithmetic average of the Voigt and Reuss bounds, is employed to isotropize the predictions. Although the VRH average model is relatively simple in form, it proves to be effective in mitigating the anisotropy present in the Voigt and Reuss models, thereby offering more accurate and reliable predictions for the elastic properties of the composite material.

(4) $$\mathrm{VRH}\_P=\frac{P_{V\;}+P_{R}}{2}$$

where P_V_ and P_R_ represent the upper and lower bound obtained by Voigt and Reuss models, respectively.

#### 3.1.3. The Isotropized Voigt–Reuss (Iso-VR) Model [23]

The Isotropized Voigt–Reuss model is specifically designed to address the anisotropy inherent in the Voigt and Reuss models, based on equivalence of strain energy [23]:

(5) $$\mathrm{Iso}\text{-}\mathrm{VR}\_P=\frac{2}{\frac{1}{P_{V}}+\frac{1}{P_{R}}}$$

where P_V_ and P_R_ are elastic and shear moduli calculated by the Voigt and Reuss formulae, respectively.

#### 3.1.4. The Generalized Self Consistent (GSC) Model [10]

The generalized self-consistent model is the improvement of the self-consistent model, which considers a model with a single inclusion embedded first with a matrix and followed by an infinite composite phase of unknown properties. This consideration accounts for the interaction between inclusions, overcoming the limitations of the self-consistent model. The complex analytical solutions obtained for the calculation of shear modulus and bulk modulus are given below:

(6) $${A\left(\frac{\mathrm{GSC}\_G}{G_{1}}\right)}^{2}+2B\left(\frac{\mathrm{GSC}\_G}{G_{1}}\right)+C=0$$

(7) $$\mathrm{GSC}\_K=K_{1}+\frac{f_{2}(K_{2}-K_{1})}{1+\frac{f_{1}(K_{2}-K_{1})}{K_{1}+\frac{4}{3}G_{1}}}$$

where

$$A=8\left(\frac{G_{2}}{G_{1}}-\;1\right)\left(4-5\nu_{1}\right)\eta_{1}f_{2}^{\frac{10}{3}}–\;2\left(63\left(\frac{G_{2}}{G_{1}}-\;1\right)\eta_{2}+2\eta_{1}\eta_{3}\right)f_{2}^{\frac{7}{3}}+252\left(\frac{G_{2}}{G_{1}}-\;1\right)\eta_{2}f_{2}^{\frac{5}{3}}-\;50\left(\frac{G_{2}}{G_{1}}-\;1\right)(7-\;12\nu_{1}+8{\nu_{1}}^{2})\eta_{2}f_{2}+4(7-\;10\nu_{1})\eta_{2}\eta_{3}\;B=-2\left(\frac{G_{2}}{G_{1}}-\;1\right)\left(1-5\nu_{1}\right)\eta_{1}f_{2}^{\frac{10}{3}}+2\left(63\left(\frac{G_{2}}{G_{1}}-\;1\right)\eta_{2}+2\eta_{1}\eta_{3}\right)f_{2}^{\frac{7}{3}}-\;252\left(\frac{G_{2}}{G_{1}}-\;1\right)\eta_{2}f_{2}^{\frac{5}{3}}+\;75\left(\frac{G_{2}}{G_{1}}-\;1\right)(3-\nu_{1})\eta_{2}\nu_{1}f_{2}+\frac{3}{2}(15\nu_{1}-\;7)\eta_{2}\eta_{3}\;C=4\left(\frac{G_{2}}{G_{1}}-\;1\right)\left(5\nu_{1}-\;7\right)\eta_{1}f_{2}^{\frac{10}{3}}-2\left(63\left(\frac{G_{2}}{G_{1}}-\;1\right)\eta_{2}+2\eta_{1}\eta_{3}\right)f_{2}^{\frac{7}{3}}+252\left(\frac{G_{2}}{G_{1}}-\;1\right)\eta_{2}f_{2}^{\frac{5}{3}}+\;25\left(\frac{G_{2}}{G_{1}}-\;1\right)({\nu_{1}}^{2}-\;7)\eta_{2}f_{2}-(7+5\nu_{1})\eta_{2}\eta_{3}$$

With $\eta_{1}$, $\eta_{2}$, and $\eta_{3}$ given by:

$$\eta_{1}=\left(\frac{G_{2}}{G_{1}}-\;1\right)(7-\;10\nu_{1})\;(7+5\nu_{2})+105(\nu_{2}-\nu_{1}),\;\eta_{2}=\left(\frac{G_{2}}{G_{1}}-\;1\right)(7+5\nu_{2})+35(1-{\;\nu }_{2})\;\eta_{3}=\left(\frac{G_{2}}{G_{1}}-1\right)(8-\;10\nu_{1})+15(1-{\;\nu }_{1})$$

#### 3.1.5. The Mori–Tanaka (MT) Model [15]

The Mori–Tanaka method, quite different from the GSC model, evaluates the average strain of an isolated particle experiencing the average stress in an infinite matrix. The MT closed-form solutions for the calculation of effective shear modulus and bulk modulus are expressed in (8) and (9):

(8) $$\mathrm{MT}\_G=\frac{f_{2}(G_{2}-G_{1})}{1+\frac{f_{1}(G_{2}-G_{1})}{G_{1}+\frac{G_{1}(9K_{1}+8G_{1})}{6(K_{1}+2G_{1})}}}$$

(9) $$\mathrm{MT}\_K=K_{1}+\frac{f_{2}(K_{2}-K_{1})}{1+\frac{f_{1}(K_{2}-K_{1})}{K_{1}+\frac{4}{3}G_{1}}}$$

#### 3.1.6. The Iterative Isotropization of VR and HS Bounds (Itr-Iso-VR and Itr-Iso-HS) [24]

**Figure 8:**
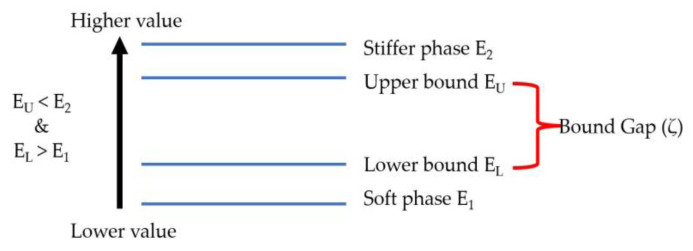
Bound gap for iterative isotropization of VR/HS model.

The Voigt–Reuss and Hashin–Shtrikman formulas are not able to obtain exact elastic properties because of the anisotropy present in the micromechanics model considered. Different attempts have been made to remove the anisotropy in the VR/HS formulas [23,25], but they are one-time isotropization and are not effective for models with strong anisotropy. As a result, the iterative isotropization model is introduced as an effective method, in which the gap between bounds is reduced iteratively by replacing the elastic moduli of the harder and softer phase with calculated upper and lower bounds, respectively, in each step. The gap (ζ) between the upper and the lower bounds is calculated using Equation (10) and illustrated in Figure 8.

(10) $$\mathrm{Bound}\;\mathrm{gap}(\zeta )=\left|\frac{\mathrm{Upper}\;\mathrm{bound}}{\mathrm{Lower}\;\mathrm{bound}}-1\right|$$

In the iterative process of the isotropized Voigt–Reuss model, there exists an initial significant gap between the upper bound (E_u_) and lower bound (E_L_) properties. To bridge this gap, the stiffer phase property (E_2_) is replaced with the upper bound (E_u_), and the softer phase property (E_1_) is replaced with the lower bound (E_L_). Subsequently, the bounds are recalculated using these newly replaced properties, leading to a reduction in the gap between them. This iterative procedure is repeated until both bounds coincide, yielding the effective properties of the composite material. Alternatively, if the required minimum bound gap is obtained during the iterations, the effective property is determined as the arithmetic average of the bounds. This approach ensures a more accurate estimation of the effective properties while effectively minimizing the anisotropy inherent in the original Voigt–Reuss and Hashin–Shtrikman models.

## 4. Results

**Figure 9:**
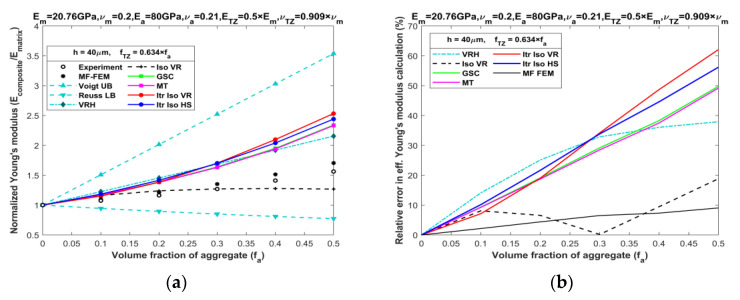
Comparison of MF-FEM and analytical models against experimental data by Yang [21]: (**a**) Young’s modulus prediction; (**b**) relative error in effective Young’s modulus calculation.

A comparison of MF-FEM and analytical predictions against the experimental data by Yang [21] is presented in Figure 9a. From the graph, it is clear that MF-FEM demonstrates a remarkable ability to closely predict the experimentally determined Young’s modulus values for the three-phase composite. Most of the micromechanics models yield unacceptable results, exhibiting a maximum error ranging between 30% to 60%, except for Iso VR, whose accuracy is not uniform. In contrast, the MF-FEM results fall well within an acceptable range, with a maximum error below 10%, as illustrated in Figure 9b.

**Figure 10:**
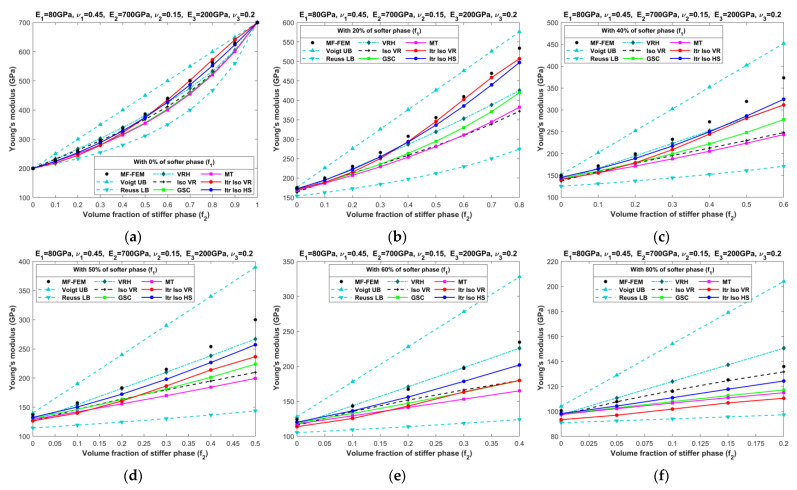
Effective Young’s moduli of SPC composites characterized by MF-FEM and analytical micromechanics models with (**a**) 0%; (**b**) 20%; (**c**) 40%; (**d**) 50%; (**e**) 60%; and (**f**) 80% of the softer phase.

**Figure 11:**
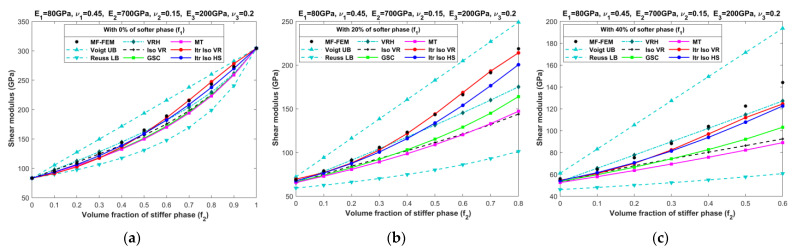

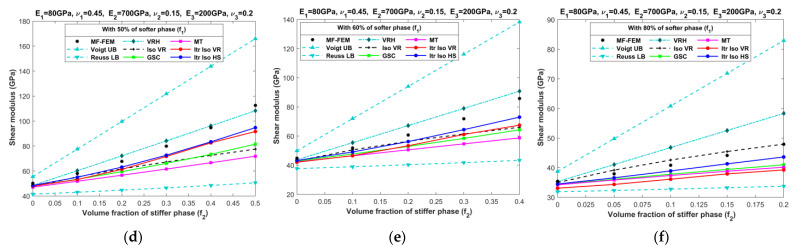
Effective shear moduli of SPC composites characterized by MF-FEM and analytical micromechanics models with (**a**) 0%; (**b**) 20%; (**c**) 40%; (**d**) 50%; (**e**) 60%; and (**f**) 80% of the softer phase.

**Figure 12:**
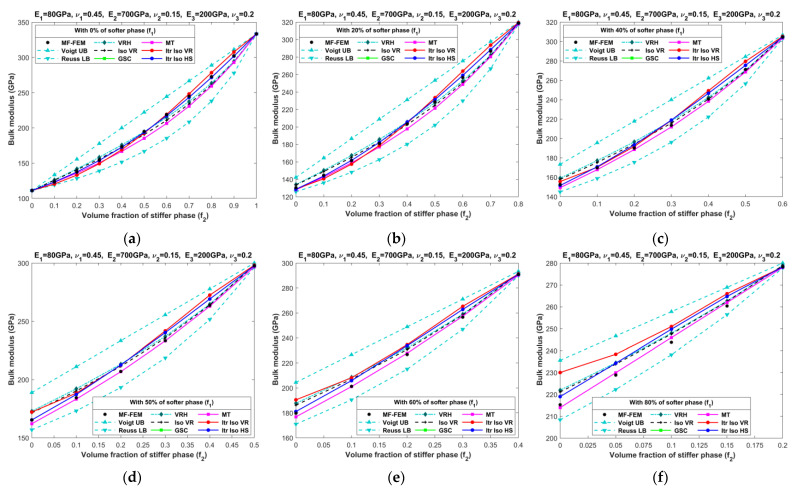
Effective bulk moduli of SPC composites characterized by MF-FEM and analytical micromechanics models with (**a**) 0%; (**b**) 20%; (**c**) 40%; (**d**) 50%; (**e**) 60%; and (**f**) 80% of the softer phase.

**Figure 13:**
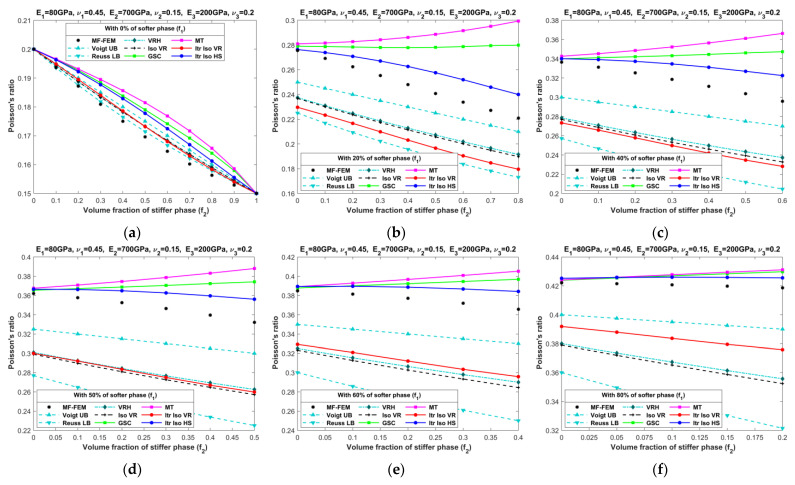
Effective Poisson’s ratios of SPC composites characterized by MF-FEM and analytical micromechanics models with (**a**) 0%; (**b**) 20%; (**c**) 40%; (**d**) 50%; (**e**) 60%; and (**f**) 80% of the softer phase.

**Figure 14:**
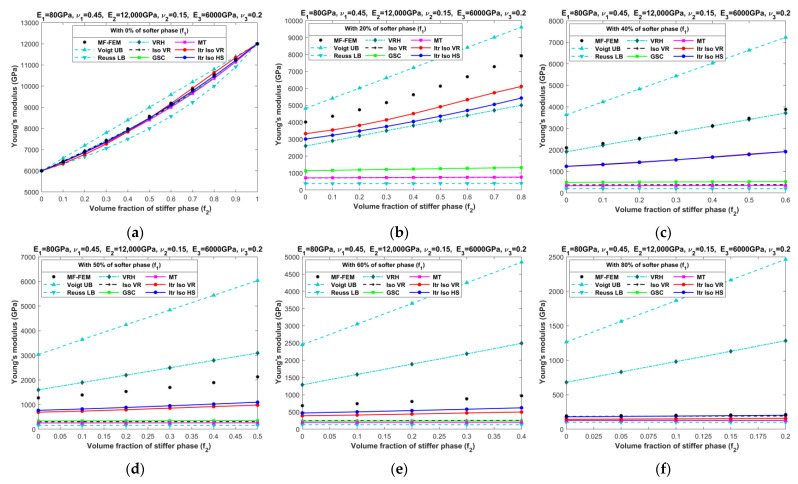
Effective Young’s moduli of LPC composites characterized by MF-FEM and analytical micromechanics models with (**a**) 0%; (**b**) 20%; (**c**) 40%; (**d**) 50%; (**e**) 60%; and (**f**) 80% of the softer phase.

**Figure 15:**
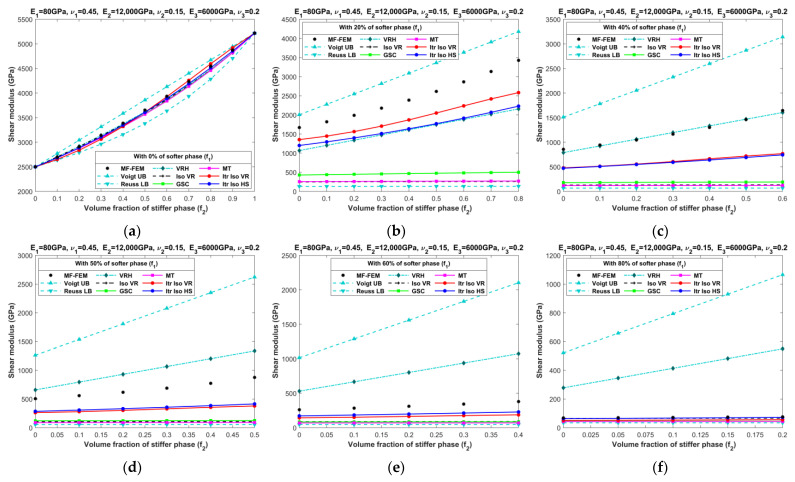
Effective shear moduli of LPC composites characterized by MF-FEM and analytical micromechanics models with (**a**) 0%; (**b**) 20%; (**c**) 40%; (**d**) 50%; (**e**) 60%; and (**f**) 80% of the softer phase.

**Figure 16:**
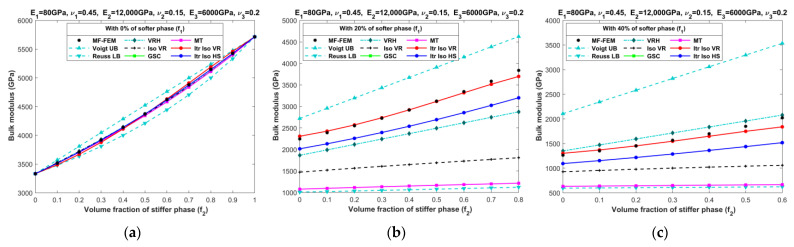

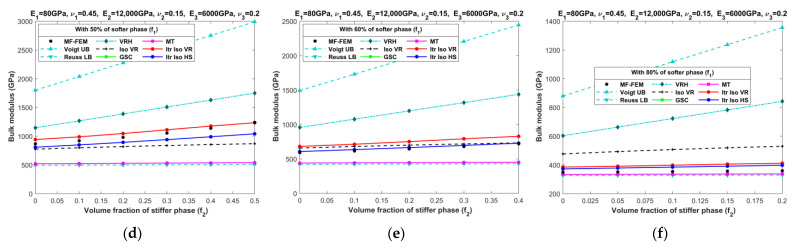
Effective bulk moduli of LPC composites characterized by MF-FEM and analytical micromechanics models with (**a**) 0%; (**b**) 20%; (**c**) 40%; (**d**) 50%; (**e**) 60%; and (**f**) 80% of the softer phase.

**Figure 17:**
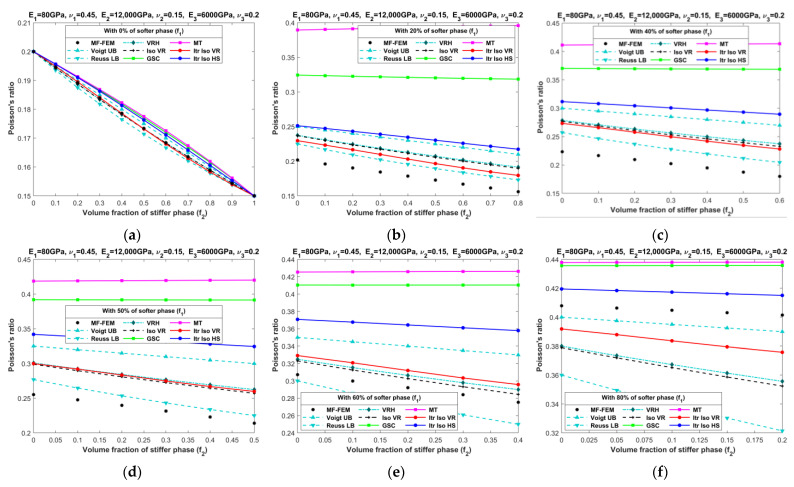
Effective Poisson’s ratios of LPC composites characterized by MF-FEM and analytical micromechanics models with (**a**) 0%; (**b**) 20%; (**c**) 40%; (**d**) 50%; (**e**) 60%; and (**f**) 80% of the softer phase.

Figure 10, Figure 11, Figure 12, Figure 13, Figure 14, Figure 15, Figure 16 and Figure 17 present the effective properties of the two types of three-phase composites, as described in Table 1, distinguished by their phase contrasts: small phase contrast (SPC) and large phase contrast (LPC). Specifically, Figure 10, Figure 11, Figure 12 and Figure 13 display the results related to SPC, while Figure 14, Figure 15, Figure 16 and Figure 17 pertain to LPC. The investigations were conducted using both MF-FEM and analytical micromechanics models. In these results, we explore the variations in effective properties while maintaining a constant volume content of the softer phase at, respectively, 0%, 20%, 40%, 50%, 60%, and 80%. Meanwhile, the volume fractions of the stiffer and intermediate phases are varied within the remaining volume. As an example, in Figure 10b, the volume fraction of the softer phase is fixed at 20%, while the remaining 80% comprises a combination of the stiffer and intermediate phases. The horizontal axis represents the volume fraction of the stiffer phase, increasing from 0% to 80%, while the corresponding volume fraction of the intermediate phase decreases from 80% to 0%.

Figure A1, Figure A2, Figure A3, Figure A4, Figure A5, Figure A6, Figure A7 and Figure A8 in Appendix A showcase the relative errors in the effective properties predicted by the analytical micromechanics models against the results obtained from MF-FEM, for both SPC and LPC composites. The relative error is defined as,

(11) $$\delta =\frac{\left|{\overset{-}{P}}_{\mathrm{MF}\text{-}\mathrm{FEM}}-{\overset{-}{P}}_{\mathrm{MM}}\right|}{{\overset{-}{P}}_{\mathrm{MF}\text{-}\mathrm{FEM}}}\times 100\%$$

The results presented in Figure 10, Figure 11, Figure 12, Figure 13, Figure 14, Figure 15, Figure 16 and Figure 17, as well as in Figure A1, Figure A2, Figure A3, Figure A4, Figure A5, Figure A6, Figure A7 and Figure A8 in Appendix A, yield several noteworthy observations. These findings collectively offer valuable insights into the accuracy of analytical micromechanics models when benchmarked against the MF-FEM results.

### 4.1. Three-Phase Composites with Small Phase Contrast (SPC)

**Figure A1:**
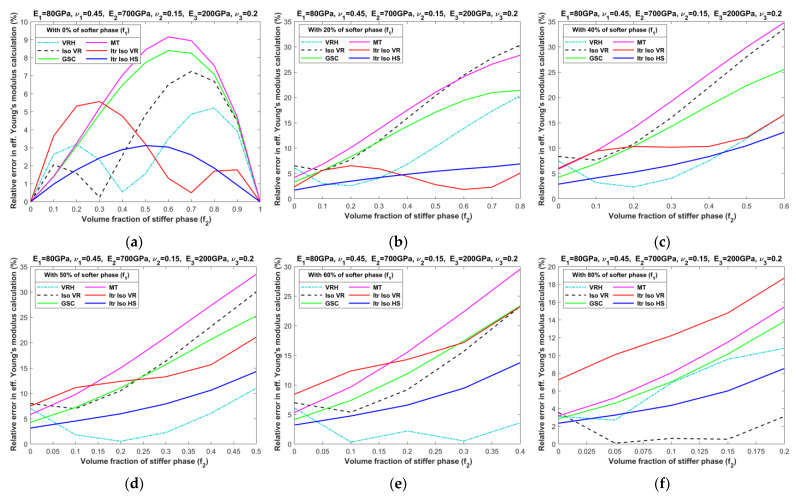
Relative errors in effective Young’s moduli of SPC composites with: (**a**) 0%; (**b**) 20%; (**c**) 40%; (**d**) 50%; (**e**) 60%; and (**f**) 80% of softer phase.

**Figure A2:**
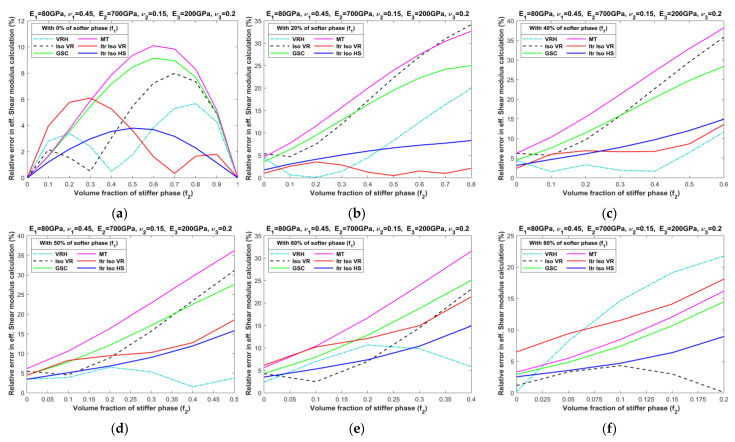
Relative errors in effective shear moduli of SPC composites with: (**a**) 0%; (**b**) 20%; (**c**) 40%; (**d**) 50%; (**e**) 60%; and (**f**) 80% of softer phase.

**Figure A3:**
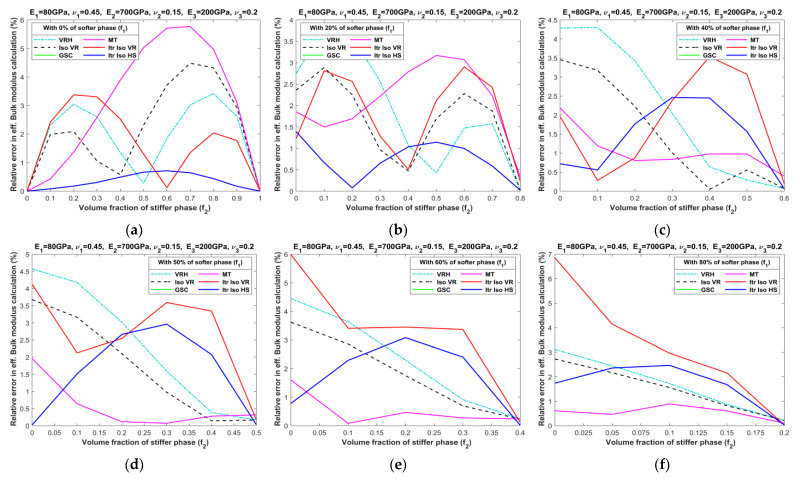
Relative errors in effective bulk moduli of SPC composites with: (**a**) 0%; (**b**) 20%; (**c**) 40%; (**d**) 50%; (**e**) 60%; and (**f**) 80% of softer phase.

**Figure A4:**
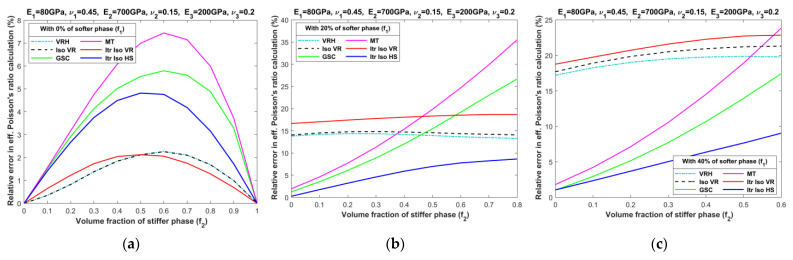

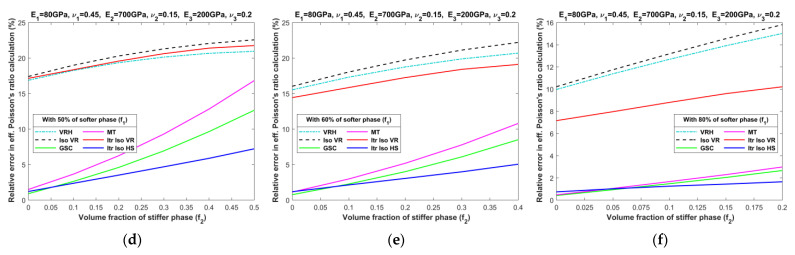
Relative errors in effective Poisson’s ratios of SPC composites with: (**a**) 0%; (**b**) 20%; (**c**) 40%; (**d**) 50%; (**e**) 60%; and (**f**) 80% of softer phase.

1. The relative errors in the effective Young’s modulus and shear modulus exhibit similar trends, while those in Poisson’s ratio and bulk modulus differ significantly.
2. Analytical micromechanics models based on Voigt and Reuss assumptions, namely VRH, Iso VR, and Iterative Iso VR, demonstrate inconsistent patterns of accuracy, with fluctuating levels of error whereas the MT, GSC, and Iterative Iso HS models consistently exhibit increased error when the volume of the stiffer phase increases.
3. Accuracy in effective Poisson’s ratio decreases with the increase in volume fraction of the stiffer phase for all models. In most cases, VRH, Iso VR, and Iterative Iso VR models initially have a relatively high error which increased slowly afterward while the remaining models have less error at the early phase that increased steeply with the increase in the stiffer phase.
4. Almost all models are able to predict the bulk modulus across a wide range of volume fractions of all three phases. The maximum error of 7% is observed with the Iterative Iso VR while the error remains below 5% for other models. Unlike other elastic properties, the contrast in the bulk modulus among phases diminishes from left to right (as seen in Figure 12) due to the chosen values for Poisson’s ratio. Eventually making the models more accurate when we increase the content of stiffer phase.
5. Overall, the Itr-Iso-HS model appears to be the most reliable analytical model for the prediction of final properties. The error in the prediction of final properties using Itr-Iso-HS is less sensitive to the volume fraction of phases compared to the remaining models as seen from Figure A1, Figure A2, Figure A3 and Figure A4.

### 4.2. Three-Phase Composites with Large Phase Contrast (LPC)

**Figure A5:**
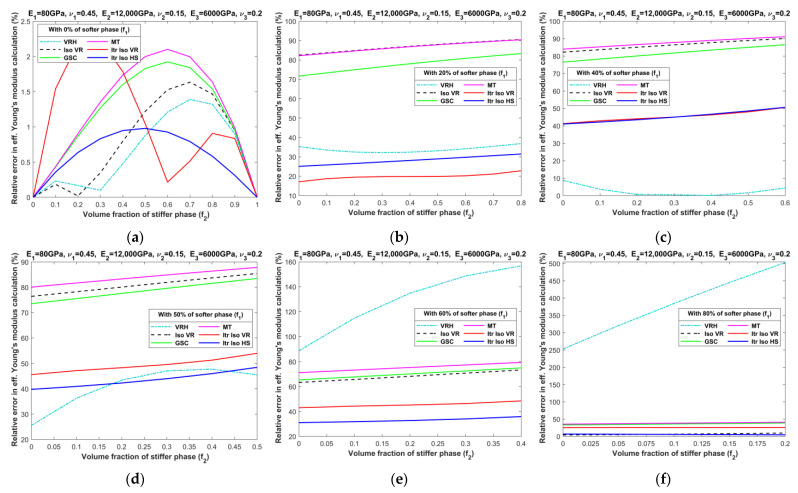
Relative errors in effective Young’s moduli of LPC composites with: (**a**) 0%; (**b**) 20%; (**c**) 40%; (**d**) 50%; (**e**) 60%; and (**f**) 80% of softer phase.

**Figure A6:**
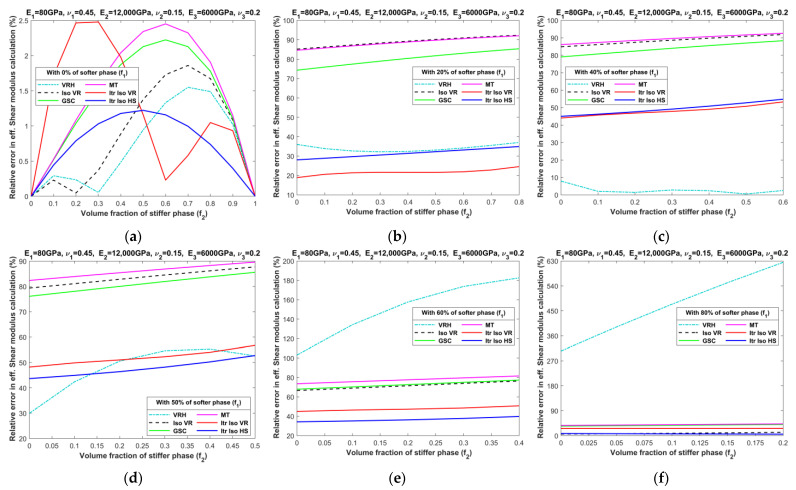
Relative errors in effective shear moduli of LPC composites with: (**a**) 0%; (**b**) 20%; (**c**) 40%; (**d**) 50%; (**e**) 60%; and (**f**) 80% of softer phase.

**Figure A7:**
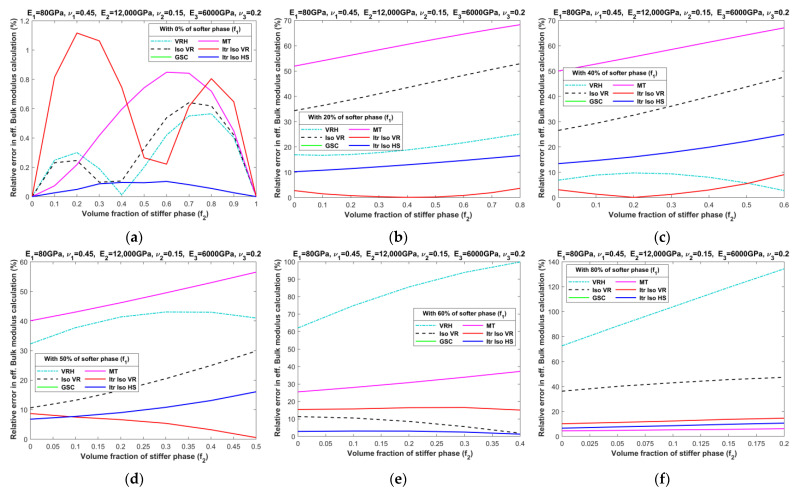
Relative errors in effective bulk moduli of LPC composites with: (**a**) 0%; (**b**) 20%; (**c**) 40%; (**d**) 50%; (**e**) 60%; and (**f**) 80% of softer phase.

**Figure A8:**
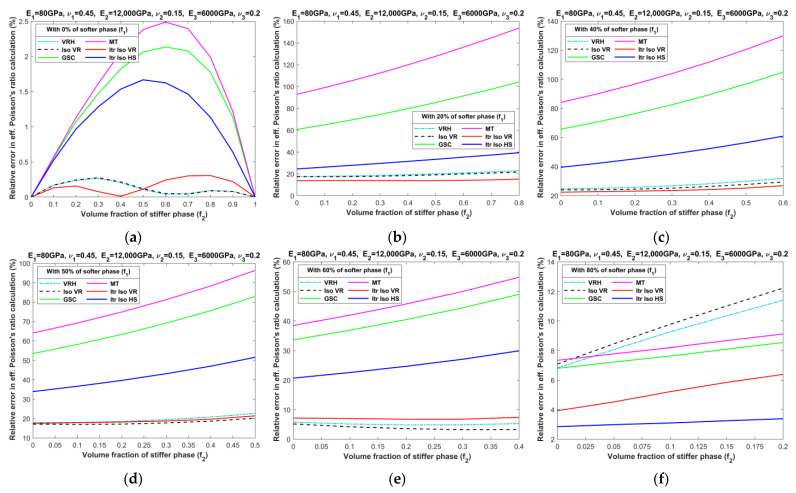
Relative errors in effective Poisson’s ratios of LPC composites with: (**a**) 0%; (**b**) 20%; (**c**) 40%; (**d**) 50%; (**e**) 60%; and (**f**) 80% of softer phase.

1. In general, the accuracy of analytical micromechanics models in predicting LPC composite properties is much lower than those of SPC composites. It should be noted that the ratio between the phase Young’s moduli of the stiffer phase to the intermediate phase is 3.5 for SPC composites, while it is 2.0 for LPC composites. This explains why the errors in Figure A1a, Figure A2a, Figure A3a and Figure A4a are larger than those in Figure A5a, Figure A6a, Figure A7a and Figure A8a, which actually correspond to two-phase composites in the absence of the softer phase.
2. For LPC composites, none of the analytical micromechanics models have acceptable accuracy in predicting the effective properties over the whole range of volume fraction. Some of them appear to be good only in a few specific cases.
3. When the volume fraction of the softer phase is high (80%), Iso HS is reasonably accurate for all elastic properties with a maximum 10% error. The Iso VR model is also in the acceptable range with an error under 12% for Young’s modulus, shear modulus, and Poisson’s ratio. However, for bulk modulus, its error again maximizes up to 47%.

## 5. Discussion

Analytical models are very easy to apply to predict the properties of composites but are only effective when there is a small mismatch in Young’s modulus among the phases and their volumetric presence is low. They cannot be implemented beyond their theoretical assumptions. The assumptions underlying micromechanical models only come into effect when the aforementioned conditions are met. For instance, in the ‘SC’ scheme, the assumption of a perfect bond between the particle and the effectively infinite medium—ensuring continuity in both displacement and traction across the interface between phases—is valid only when there is a minimal difference in the properties of the inclusions and the matrix. Similarly, the ‘GSC’ scheme is reasonable to apply within low to moderate particulate concentrations and should be aware that the final property is independent of the particle size and distribution. In the same way, the ‘MT’ method ignores the interaction among inclusions based on dilute dispersion assumptions, due to which its application on high particle-concentrated composites may lead to significant error, failing to account for particle interaction.

On the other hand, MF-FEM is more accurate and has a wide range of applicability. It can become a powerful tool in the study and design of new composite materials. Most analytical models are developed for estimating properties of the two-phase composites, and their iterative application into three or more phases will induce further errors. MF-FEM can easily be applied to multiphase composites over the whole range of volume fraction of constituents, with the necessary study on the element-to-RVE size ratio. The MF-FEM can also be the foundation to study different analytical models in the absence of appropriate experimental data.

## 6. Conclusions

The primary aim of this research is twofold: first, to validate the MF-FEM approach using experimental data on three-phase composites, and second, to conduct a numerical investigation to assess the predictive capability of analytical models using MF-FEM as a benchmark. The scarcity of experimental data for three-phase composites limits the validation of analytical models across the entire range of phase volume fractions, thereby underscoring the importance of employing MF-FEM for this purpose. Our convergence study reveals that the element-to-RVE size ratio for three-phase particulate composites is substantially smaller than that for their two-phase counterparts—specifically, 1/150 as opposed to 1/50. Validation against two independent sets of experimental data for three-phase composites confirms the reasonable accuracy of the MF-FEM approach, with a maximum error margin below 10%. Furthermore, we use MF-FEM results to evaluate the performance of various micromechanical models in the context of three-phase composites. Our findings indicate inconsistent accuracies and heterogeneous performance trends among these models. Specifically, analytical models demonstrate reasonable accuracy for LPC composites when the volume fraction of the stiffer phase is low. However, for predicting elasticity in LPC composites, all models are inaccurate. The Iso HS model emerges as the only model with acceptable accuracy, but only when the volume fraction of the softer phase exceeds 80%. Hence, the contrast in the properties between different phases acts as a critical determinant for the accuracy of analytical models.

## Figures and Tables

**Table 1:** Material properties and volume content of phases.

Phase Types	Small Phase Contrast (SPC)		Large Phase Contrast (LPC)		Volume Fraction (%)
	Young’s Modulus (GPa)	Poisson’s Ratio	Young’s Modulus (GPa)	Poisson’s Ratio	
Softer phase	80	0.45	80	0.45	30
Stiffer phase	700	0.15	12,000	0.15	35
Intermediate phase	200	0.2	6000	0.2	35

**Table 3:** Material Properties of epoxy and sand.

Properties	Epoxy	Sand
Young’s modulus—E(GPa)	2.03	73.6
Poisson’s ratio—ν	0.4	0.25
Density—ρ (g.cm^−3^)	1.1	2.63

**Table 4:** Elastic properties of mortar phases (thickness of TZ, h = 40 μm) [21].

Properties	Cement Paste	Transition Zone (TZ)	Aggregate
Young’s modulus—E (GPa)	20.76	0.5 × E_m_	80
Poisson’s ratio—ν	0.2	0.909 × ν_m_	0.21

**Table 5:** The measured elastic modulus of mortar [21].

Designation	*f*_a_ (%)	*f*_TZ_ (0.634 × *f*_a_) (%)	E_c_ (GPa) [21] * (Measured)
M0	0	0	20.760
M10	10	6.34	22.304
M20	20	12.68	24.141
M30	30	19.02	26.350
M40	40	25.36	29.292
M50	50	31.7	32.439

* Average of three specimens.

## Data Availability

Not applicable.

## Appendix A. Relative Errors in Effective Elastic Properties Predicted by the Analytical Micromechanics Models against Results Obtained by MF-FEM

Figure A1, Figure A2, Figure A3 and Figure A4 show the relative errors related to SPC composites, while Figure A5, Figure A6, Figure A7 and Figure A8 presents those pertaining to LPC composites.
